# Facilitators and barriers to healthy food selection at children’s sports arenas in Norway: a qualitative study among club managers and parents

**DOI:** 10.1017/S1368980020003985

**Published:** 2021-04

**Authors:** Lisa Garnweidner-Holme, Hilde Sørfonn Haugland, Ingrid Joa, Vibeke H Telle-Hansen, Gyrd Omholt Gjevestad, Mari CW Myhrstad

**Affiliations:** 1 Department of Nursing and Health Promotion, Faculty of Health Sciences, Oslo Metropolitan University, P.O. 4, St.Olavs Plass, Oslo 0130, Norway; 2 TINE SA, Oslo, Norway

**Keywords:** Sports arenas, Handball, Healthy food, Children, Qualitative research

## Abstract

**Objective:**

To investigate club managers’ and parents’ experiences with food selection at handball halls in order to identify facilitators and barriers to the availability of healthy food.

**Design:**

Individual interviews with club managers (*n* 6) and focus groups (*n* 5) with parents (*n* 21) were conducted. Interviews were recorded and transcribed verbatim, transcripts were coded in NVivo and the analysis was guided by thematic analysis.

**Setting:**

Interviews were conducted at five handball clubs with varying socio-economic user populations and sizes in the area of Oslo, Norway.

**Participants:**

The club managers were responsible for food selection at the handball clubs. The participating parents had one or two active children between the ages of 6 and 12 years who took part in the clubs.

**Results:**

The club managers and parents generally described food selection at the handball halls as unhealthy and wanted a healthier selection of food. The club managers’ primary barriers to providing a healthier food selection included the potential to lose profits, limited facilities and time to prepare these foods. The parents often valued unhealthy food, as they believed that it supported the social environment and served as a reward for the children. Trainers were perceived as important role models for the promotion of healthy eating. The participants thought that national guidelines could facilitate healthy food environments in sports arenas.

**Conclusion:**

Healthier food options in sports settings could be facilitated through national guidelines that describe healthy foods and establish who is responsible for providing healthy food selections.

Healthy dietary habits established early in life often persist into adulthood and promote lifelong health^(1,2)^. A food environment that markets and increases the availability of unhealthy, energy-dense and nutrient-poor food is one of the most significant factors that contributes to unhealthy diets, obesity and non-communicable diseases in both children and adults^(3)^. Studies have documented a positive relationship between energy-dense diets and children becoming overweight^(4,5)^, and one out of three children is overweight or obese in European countries^(6)^. The prevalence of overweight and obese children appears to have reached a plateau in several European countries^(7)^. However, dietary intake and BMI clearly differ depending on socio-economic status (SES). Children and adolescents from lower SES families often have an unhealthy diet^(8,9)^ and have a higher prevalence of becoming overweight or obese^(10,11)^.

Sports are integral to many children’s lives. From a public health perspective, sports arenas are ideal for promoting health and positive healthy behaviours^(12–15)^. Children who participate in sports frequently spend considerable amounts of time in these activities outside of school. In Norway, 508 000 children between 6 and 12 years of age, including children from lower SES families, participate in organised sports activities^(16)^. Children from lower SES families tend to participate in sports activities that do not involve expensive equipment, such as football and handball^(17)^.

The influence of food selection at sports arenas on children’s dietary habits remains unclear^(18,19)^. Sports arenas, where children participate in sport, are often characterised by unhealthy food environments that offer a wide range of energy-dense, nutrient-poor, processed foods that are quick to prepare and inexpensive to provide^(12,16,20,21)^. Few studies have systematically examined the types of food and beverages that children consume during sporting events^(22)^. Studies in the United States and Switzerland have determined that convenience foods high in energy and sugar are made readily available in sports settings for youths^(23,24)^. A systematic review investigating children’s and parents’ opinions of the sport-related food environment suggests that many children and parents consider the environment neither conducive to nor supportive of children’s healthy food behaviours^(13)^.

An emerging body of the literature shows that time constraints and low access to nutritious foods are barriers for healthy eating for children at the sport arenas^(14,15,20,25)^. The provision of food at sports arenas varies depending on the type of sports arena and the country where the arena is located^(13,20,26)^. In Norway, handball arenas often have kiosks that are only occasionally opened depending on the size of the club. While club managers are the general managers of the club and mainly responsible for the kiosks and food selections, parents help out in food preparation and sales during opening hours. The profits from the kiosks are one of the three most important sources of income for sports clubs^(17)^.

The current study aimed to gain insight into the facilitators and barriers to the availability of healthy foods in children’s sports settings. Handball clubs were chosen as the current study’s settings because handball is popular in Norway and their food environments remain the same throughout the year, regardless of season or weather.

## Methods

### Sampling and participants

Club managers (*n* 6) and parents (*n* 21) from five handball clubs participated in the study. Individual interviews (*n* 6) were conducted with the club managers, and the parents were interviewed in focus groups of four to five people. A total of five groups were interviewed. One club manager (club 2) participated in both an individual interview and a focus group interview. Clubs were purposively selected from a list that one author (XX) provided. These included clubs that had no known collaboration with the food industry. Club managers were recruited by two authors (XX) via e-mail. Five out of six of the contacted clubs were willing to participate. A convenience sample of parents was recruited with the help of the club managers. In two clubs, parents were directly recruited by two authors (XX) while the children played handball. Parents had one or two children between 6 and 12 years of age in the participating clubs. Table 1 presents background information about the participating handball clubs and the gender and number of participants in each focus group. The club managers were responsible for the management of the kiosks in each club. The clubs served families with divergent ethnic and socio-economic backgrounds. The eastern region of Oslo is characterised by a higher immigrant and lower SES population than that of the western region. Participants gave their written informed consent. The study was approved by the Norwegian Center for Research Data (Project number 390687). Recruitment was carried out until we observed replication in the responses and no new themes emerged from the interviews^(27)^.

**Table 1:** Background information about the participating handball clubs and focus groups

Club	Size*	Regions in Oslo	Genders of participating club managers	Genders of participating parents	Kiosks’ opening times
1	Medium	East	1 woman	4 women	Once a week (Sunday)
2	Small	West	1 woman	4 women 1 man	Every weekend
3	Large	West	2 women	3 women 1 man	Almost every weekend
4	Large	East	1 man	3 women 1 man	Every weekend
5	Medium	East	1 woman	4 women	Once a week (Saturday)

* The clubs were categorised as small clubs (< 100 members), medium clubs (100–200 members) and large clubs (< 200 members).

### Data collection

All interviews were conducted at the handball clubs. A semi-structured interview guide (Appendix 1) was developed to ensure consistency across the individual interviews and focus group discussions and to allow for some flexibility within each group. The interviews started with a short introduction to the study’s aim, and the participants were informed that they would be able to withdraw their consent at any time without any reason. After the introduction, the discussion session began with open-ended questions. The main topics in the interview guide included the following: (1) experiences with food preparation and organisation at the clubs; (2) experiences with food selection at the clubs and (3), suggestions for initiatives to support healthier food selection at the clubs. The interview guide was pilot tested by two authors (XX) with the club manager and the parents at the first club. As the pilot test only led to minor changes in the interview guide, these interviews were included in the final analysis for the current study. Two authors (XX) were present at all the interviews, and either XX or XX was the interviewer. The individual interviews among the club managers were between 16 and 36 min long. The focus group interviews were between 19 and 34 min long. All interviews were digital audio recorded and transcribed verbatim by two authors (XX). Two additional authors (XX) read through the transcribed interviews. Interviews were conducted in Norwegian. Subthemes, overarching themes and quotes were translated into English by the authors for the purpose of the current study.

### Analysis

The analysis was guided by Braun and Clarke’s thematic analysis and included the following steps^(28)^: (1) becoming familiar with the data by repeatedly reading each informant’s transcript; (2) generating initial codes (words or short phrases in the transcripts) that were relevant to the research questions; (3) organising codes into subthemes; (4) arranging subthemes into overarching themes and (5) defining and naming the themes. Three authors (XX) conducted the analysis and discussed potential codes and themes with the other authors. The qualitative software programme NVivo (12·1) was used to identify codes and systematise subthemes.

## Results

### Descriptions and organisation of food sold at kiosks

The club managers and parents described a high level of unhealthy food selection, characterised by sausages, toast (with white bread, cheese and ham and without vegetables), waffles, cakes and chocolate, in every handball hall. They believed that their food selection options were similar to those in other clubs and healthy foods were less available at sports arenas. In this context, several participants observed that sports arenas are often associated with unhealthy food. The following statement by a club manager illustrates this sentiment:

‘Yeah … handball is known for waffles. And I think that’s a bit sad. Handball is now associated with the smell of waffles. And we sell a lot of them’ (Club 3’s manager).

Club managers said that the range of foods they offered was better (e.g. warm meals, like soups and hamburgers) and healthier (e.g. sandwiches and fruits) at big events when parents brought along a cooker. Although the club managers believed that parents and their children were satisfied with the food selections at their clubs, the parents were of the opinion that the food selections were limited and unhealthy. The club managers were primarily responsible for the kiosks and food selections, and the parents helped prepare and sell the food during opening hours. According to the managers, the profits from the kiosks were one of the primary sources of the clubs’ income. None of the participating clubs reported receiving sponsorships from the food industry.

### Facilitators and barriers to food selection at handball arenas

Table 2 summarises the facilitators and barriers to healthy food selection at the handball arenas identified by the club managers and parents. Illustrative quotes are presented below in the presentation of the results.

**Table 2:** Facilitators and barriers to healthy food selection

Main themes	Facilitators of a healthier food selection	Barriers to a healthier food selection
Sub-themes	Club managers’ and parents’ desire for healthier and more varied food selections.	Club managers’ fears of losing profits by selling healthy foods.
	Parents’ experiences in influencing food selection.	Club managers’ and parents’ limited time to prepare healthy foods.
	Trainers’ influence over children’s diet.	Club managers concern about limited shelf life of healthy foods.
	Older children wanting healthier food.	Limited kiosk facilities for the preparation of healthy foods.
	Club managers asking for guidelines on the responsibility for and selection of healthy foods.	Parents’ appreciation of unhealthy foods at the kiosks.
	Club managers asking for educational materials to support the selection of healthy foods.	Unhealthy food from the kiosks as a reward or consolation for children.

### Facilitators of healthy food selections at handball halls

Club managers’ and parents’ desires for healthier and more varied food at the handball halls were identified as an important facilitating factor for the availability of healthier food choices. Parents in some clubs felt that they could influence the food selection and decide what to sell when they were responsible for the kiosks. However, the analysis revealed that most of the participants had little experience in providing healthy foods at the handball halls. In all the clubs, parents often provided their children with food from home so that they could avoid unhealthy food from the kiosks. One club manager said that it was easier to offer and sell healthy food (e.g. sliced fruits and vegetables in small boxes) at bigger events with more spectators than at smaller events. Club managers and parents at all the clubs believed that trainers’ (similar to coaches) awareness of healthy eating influenced what children ate. One club manager observed that some trainers asked the children to bring their own food instead of buying unhealthy food from the kiosks. She said:

‘They [the children] know that they can’t go to the kiosk and buy some food in between the matches, and they know what they are allowed to eat. Because they are very concerned about what is right to eat, and they are not allowed to buy sweets or something like that’ (Club 1’s manager).

Children’s awareness of healthy eating appeared to influence their food selection at the kiosks. The parents noted that younger children wanted to purchase unhealthy foods at the handball halls more than older children. The club managers and parents observed that older children were more concerned about eating healthy. As one participant expressed:

‘Yes, I don’t really know if the younger girls are so concerned about it. They are very concerned to get their amount of sweets and soft drinks. But I think that the older girls – I mean those who are 13–14 years old—they are concerned about healthy eating’ (Parent 2, club 5).

Club managers and parents believed that guidelines from the government or sports confederations about the types of food that should be offered at sport arenas could support the selection of healthier food. A parent from one of the larger clubs said:

‘No, I think that if you want to change it [the unhealthy food selection] in general, for handball, there have to be some guidelines. So, for example, the handball confederation should come with guidelines for the kiosks. And implement them. Promote them at different occasions’ (Parent 1, club 3).

Club managers emphasised that these guidelines should also define who is primarily responsible for providing healthy food selections at the clubs. The club managers and parents also asked for educational materials to provide information about healthier alternatives to fast food and cakes. The club managers proposed the need for informational materials that could motivate parents to prepare healthier foods. The club managers further suggested the need to collaborate with retailers and food industries to make their food selections healthier.

### Barriers to healthy food selection at handball halls

Losing profits was a significant hindrance to selling healthier foods at all clubs independent of the characteristic SES of their user population. In support of this idea, a club manager said:

‘No, the challenge is that we lose profits. We just have to find something that is healthier that brings us as much money’ (Club 3’s manager).

The same participant also mentioned that they lacked time to prepare healthy food, either at home or at the handball hall:

‘And it is also challenging because none of us has enough time to prepare it [healthy food]. Otherwise, I don’t see any challenges. I would have been proud if my club would be a role model for both healthier waffles and cakes’ (Club 3’s manager).

According to the club managers, the limited shelf life of healthy foods made it challenging to offer them, regardless of the size or characteristic SES of the user population of the clubs. All the clubs depended on foods with long shelf lives since most of them only opened the kiosk every weekend or every second or third weekend. The club managers and parents also observed that healthier foods created more food waste.

The club managers and parents also believed that the handball halls’ kitchen facilities were too limited for the provision of healthy food. None of the clubs had a microwave oven or a cooker. As a parent at a medium-sized club noted:

‘It’s only a worktop. You raise a shield and then it’s open with a bank behind and a coffee machine and nothing else. And a fridge is there. There is no wash, so if you need water, you have to go to the awful changing room. There is no place to prepare food’ (Parent 2, club 5).

Another barrier to the selection of healthy food included the parents’ attitudes about food at the handball halls. Some parents were of the opinion that their children could eat unhealthy food at the handball halls since it would not influence their overall diets. As a parent at a club where the kiosk was opened almost every weekend expressed:

‘I think you should have a pragmatic view on it. When people are active, children are active, so if they sometimes eat chocolate, it does not matter. Or chips. Don’t get hysterical because it could lead them into the opposite direction. If there is a handball match once a week, and children eat unhealthy on that one occasion, that’s not the foundation for an unhealthy diet. It’s what children eat each day that’s the most important’ (Parent 1, club 3).

The parents often appreciated eating unhealthy food with the other parents at the handball halls. The club managers and parents also stated that unhealthy food from the kiosks often served as a reward and consolation for the children. As a club manager described it:

‘I have a feeling that for many, both children and parents, the sports arena is a pleasurable time for them. There you should get some award after a match, and two or three … hmm … and that you get something that you usually don’t get. So hat what you buy at the sports arena is an award for good effort’ (Club 4’s manager).

## Discussion

The selection of food at the Norwegian handball clubs was described as predominately unhealthy. Although the club managers and parents wanted a more varied and healthy food selection, emerging barriers to healthy food selection included lost profits from selling healthy foods and limited time and facilities to prepare it. While the parents asked for more healthy food, they concluded that unhealthy food at the handball halls held social importance, both for themselves and as a reward for their children. Trainers were perceived as important role models for the promotion of healthy eating. Initiatives that the participants suggested for the provision of healthier food selections included national guidelines about the kind of healthy foods that can be offered at sports arenas and educational materials for how to prepare them.

### Parents’ ambivalence about healthy food options at sports arenas

Parents play an important role in forming and supporting children’s healthy dietary habits^(29)^. In the present study, the participating parents asked for more varied and healthy food. Nonetheless, they valued unhealthy food because they believed that it supported the social environments of the handball halls. Similar findings were determined in a focus group study among parents of children who participated in basketball programmes in Minnesota^(25)^. While some parents were opposed to unhealthy snacks at the basketball arena, others were less concerned about them. In line with the current study, many of these parents viewed unhealthy foods as an occasional treat^(25)^. Most of the kiosks in our study were only opened once a week, and the parents did not think that occasional provision of unhealthy food negatively impacted their children’s diet.

The impact that food selections at sports arenas have on children’s dietary habits remains unclear^(18,19)^. Parents in the current study often used unhealthy foods at the handball kiosks as rewards for their children. A Norwegian cohort study found that the parental use of food as a reward for 6-year-old children predicated emotional overeating 2 years later^(30)^. During the present study’s interviews, club managers perceived that handball trainers appeared to be more aware of the consequences of what the children were eating than their parents. We have not found any studies specifically investigating the trainers’ roles in childhood food consumption in sports settings. However, a review of the effectiveness of family-based and institutional interventions in improving children’s diets determined that effective school-based programmes should incorporate role models such as peers, teachers and heroic figures, in addition to rewards and increased access to healthy foods^(31)^. Thus, the trainer may have an important role in promoting healthy eating habits.

### Feasibility of healthy food selections at sports arenas

Sports arenas may be useful settings for promoting health^(12–15)^. An integrative review, where key issues about youth sport clubs as health-promoting settings were identified, concludes that sport clubs need to provide activities designed for, and adapted to, the specific age group or stage of the development of the youth^(14)^. In Norway, children of lower SES families attend sports activities and take part in sports that do not involve high expenses for equipment, such as handball. These arenas are considered to be important environments for reducing social disparities related to healthy eating^(17)^. We identified similar barriers for the feasibility of healthy food selections at sports arenas in clubs with members from both low and high SES families, such as profit loss and limited time and kitchen facilities for the preparation of healthy foods. Thus, limited resources of the sport clubs is an eminent barrier for a healthy food provision. This is also shown to be an important barrier in other countries^(15)^. In contrast to studies in Australia, United States and New Zealand, sponsorship from unhealthy food producers did not appear as a barrier for a healthy food provision in the participating clubs in our study^(22,32,33)^. This might be because none of our participating sport clubs received any sponsorship. Parents in our study often prepared the food they sold at the kiosks at home. As such, they believed that some structural barriers to preparing healthy food at handball halls could be overcome through the provision of educational materials (e.g. recipes to make unhealthier foods healthier).

### Guidelines for healthier food environments at sports arenas

The club managers and parents noted that guidelines by national authorities or sports confederations could support healthy food selections at sports arenas. In an Australian telephone survey, most parents agreed that the government should restrict the sale of unhealthy foods and beverages at children’s sporting venues^(21)^. The present study’s participants did not express the desire for policies to restrict unhealthy food. Rather, they asked for practical guidelines on the kinds of healthy foods that should be offered and how to prepare them. There is general agreement that policies and guidelines can support healthier food environments^(34,35)^. Thomas et al. suggested practice recommendations to promote healthy eating among youth sports participants^(25)^. In line with the current study’s results, these recommendations endorsed broader collaboration between sports clubs, parents, food retailers and public health professionals. Thomas et al. also suggested the development of user-friendly educational materials about nutrition for parents, coaches and children to inform them about the types of food that are appropriate for sports settings. It has to be acknowledged that there is little evidence regarding the effectiveness of strategies to improve the implementation of policies, practices or programmes for healthier food provision at sporting clubs^(36)^. A prospective study assessing whether voluntary nutrition guidelines had an impact on food environments in recreation and sports settings in a Canadian province found that voluntary guidelines alone were insufficient at improving the food choices available for children^(12)^. However, significant improvements in facility capacity, nutrition policy and food environment quality occurred in sport and recreation facilities that were exposed to the nutrition guidelines^(37)^.

## Study limitations

The current study was conducted among a small sample size, which is typical of qualitative studies^(28)^. Nonetheless, we believe that our findings may be transferable to handball clubs with similar sizes, user groups, economies and facilities. However, it has to be acknowledged that the researchers’ professional backgrounds and personal experiences might have shaped the gathering and interpretation of data. Even though recruitment was carried out until we observed replication in the responses^(27)^, interviewing a more diverse range of parents, in terms of sports, locations or demographic backgrounds, might have revealed additional themes. It is also worth noting that sports clubs in larger cities in Norway are wealthier than in the country’s rural areas^(17)^. In addition, while we did not collect the demographic characteristics of the study’s participants, their ethnic and socio-economic backgrounds were representative of the user profile of the members of the handball clubs. Most of the participants were mothers, which might have influenced the results of the current study, as mothers tend to be more concerned about healthy eating than fathers^(38)^.

## Conclusion

The club managers and parents described a predominantly unhealthy selection of food at the handball halls. Although they wished for a more varied and healthier food selection, we identified individual barriers for a healthier food environment at the handball hall. For instance, parents valued unhealthy food because they believed that it supported the social environments of the handball halls. The present study’s interviews also revealed several structural barriers, such as profit lost and limited kitchen facilities, that must be overcome for healthier food to be provided at the participating handball clubs. Healthier food options in sports settings could be facilitated through national guidelines that describe healthy foods and establish who is responsible for providing healthy food selections. The trainer may have an important role in the promotion of healthy eating at the sports arenas.

## Supplementary material

For supplementary material accompanying this paper visit https://doi.org/10.1017/S1368980020003985. click here to view supplementary material
