# Supplementary material for: Facilitators and barriers to healthy food selection at children’s sports arenas in Norway: a qualitative study among club managers and parents
Source: Public Health Nutr. 2020 Oct 12;24(6):1552–8. doi: 10.1017/S1368980020003985 (PMC8025096; doi:10.1017/S1368980020003985)
Supplement: Supplementary file 1 [file S1368980020003985sup001.docx]

**Interview guide individual interviews club managers**

**Introduction**

- 1. Welcoming and introduction of the researchers
  2. Information about the aim of this interview
  3. Information about participant’s privacy and obtaining written informed consent

**Main topics and probing questions**

2.1 General introduction about food at the sports arena

a) Can you describe your duties in the club and how long you have been working in the club?

b) Can you explain your responsibilities and duties related to the food selection at the club?

c) What do you think about the food selection at the sports arena in general, and in your club?

2.2 Experiences with the food at the club

a) What are your experiences with the preparation of the food at your club? What works and what does not work?

b) What do you think influences children’s food intake at the sports arena?

c) To what extent are parents involved in the food preparation at your club?

d) Do you have any informational material related to the food preparation at your club?

3.3 Today’s food selection at the club

a) Can you describe the food selection at your club?

b) Can you describe how the kiosk is organized?

c) How do you think parents perceive the food selection at your club?

3.4 Initiatives for a healthy food selection at the club

a) Do you have any suggestions for how to facilitate a healthy food selection at the club?

b) Are you willing to imply them?

c) Who do you think should be responsible for a healthy food selection at the sports arena?

**Conclusion**

4.1 Clarifying questions

4.2 What was your motivation to participate in this study?

4.3 Thanking participants for their involvement

**Interview guide focus group interview parents**

**Introduction**

- 1. Welcoming and introduction of the researchers
  2. Information about the aim of this interview
  3. Information about participant’s privacy and obtaining written informed consent

**Main topics and probing questions**

2.1 General introduction about food at the sports arena

a) How many of your children are active in the club and can you describe your children’s activities in the club?

b) What do you think about the food selection at the sports arena in general, and in your club?

2.2 Experiences with the food at the club

a) Have you been involved in the food serving at the kiosk in the club? If yes, can you explain your responsibilities and duties related to the food selection at the club?

b) Did you receive any informational material related to the food preparation at your club?

3.3 Today’s food selection at the club

a) Can you describe the food selection at your club?

b) Can you describe how you experience this selection?

c) Can you tell us what you use to buy at the kiosk, both for yourselves and your children?

d) Do you perceive that you can influence the food selection at the kiosk?

3.4 Initiatives for a healthy food selection at the club

a) Do you buy healthy food at the kiosk?

b) Do you have any suggestions for how to facilitate a healthy food selection at the club?

c) Who do you think should be responsible for a healthy food selection at the sports arena?

**Conclusion**

4.1 Clarifying questions

4.2 What was your motivation to participate in this study?

4.3 Thanking participants for their involvement

Vedlegg 3: Intervjuguide fokusgruppeintervjuer med foreldre

**Steg 1: Introduksjon**

Informasjon om formål med intervjuet og personvern, samt spørsmål om muligheten for å kunne ta kontakt med klubbledelse i etterkant av studien (jf. informasjonsskriv).

**Formål:** Det overordnede målet med dette prosjektet er å få en bedre en forståelse for faktorer som kan påvirke mattilbudet og identifisere mulige tiltak for å fremme sunn og god mat på idrettsarenaen.

Det er frivillig å delta i prosjektet. Hvis du velger å delta, kan du når som helst trekke samtykke tilbake uten å oppgi noen grunn. Det vil ikke ha noen negative konsekvenser for deg hvis du ikke vil delta eller senere velger å trekke deg. Intervjuene vil tas opp på lydopptaker, og all data vil bli anonymisert, slik at det ikke registreres opplysninger i prosjektet som gjenkjenner enkeltpersoner

Prosjektet skal etter planen avsluttes høsten 2022. Lydfiler og all data vil da bli slettet.

Forskningsprosjektet er godkjent av Norsk senter for forskningsdata (NSD), og er et forskningssamarbeid mellom OsloMet – storbyuniversitetet og TINE SA.

**Steg 2: Intervju**

Problemstilling: *Hva er mulige faktorer som kan påvirke et sunt mattilbud på idrettsarenaen?*

**1. Tema: Generell introduksjon om mat på idrettsarenaen**

- • Fortell litt om deres barns deltakelse i klubben. Hvor mange barn har dere som er aktive og i hvilken alder er de?
- • Hva tenker dere rundt maten som tilbys på idrettsarrangementer generelt og i deres klubb?

**2. Tema: Erfaring fra matservering i klubben**

- • Kan dere fortelle oss om dere har vært involvert i matverseringen? Hvis ja – til hvilke anledninger?
- • Fikk dere noe veiledningsmateriell for å lage mat ifm. dette? Hvis ja – kan dere fortelle oss om deres erfaringer med dette?

6 2

**3. Tema: Dagens tilbud i klubben**

- • Kan dere fortelle litt om matutvalget som tilbys i klubben til vanlig og under idrettsarrangementer. Hva serveres? Pris?
- • Hvordan opplever dere dette tilbudet?
- • Er det noe dere savner ved dagens tilbud?
- • Hva kjøper dere til dere og til barna? Er det en forskjell? Og hvorfor kjøper dere det?
- • Hva skal til for at dere velger de sunnere alternativene? Er tilgjengelighet, pris og utvalg viktig?
- • Opplever dere at dere som foreldre har mulighet til å påvirke mattilbudet?

**4. Tema: Tiltak for et sunnere tilbud i klubben**

- • Har dere noen forslag til tiltak dere mener kunne blitt gjort for å oppnå et sunnere mattilbud i klubben?
- • Ser dere noen utfordringer med å gjøre mattilbudet sunnere?
- • Hvem tenker dere er ansvarlig for å fremme god og sunn mat på idrettsarenaen?

**Steg 3: Oppsummering**

- Hva var motivasjonen deres til å være med i studien?

- Er det noe dere ønsker å legge til? 6 3

Vedlegg 4:
